# Melatonergic agents influence the sleep-wake and circadian rhythms in healthy and psychiatric participants: a systematic review and meta-analysis of randomized controlled trials

**DOI:** 10.1038/s41386-022-01278-5

**Published:** 2022-02-04

**Authors:** Eunsoo Moon, Timo Partonen, Serge Beaulieu, Outi Linnaranta

**Affiliations:** 1 Department of Psychiatry, Pusan National University School of Medicine, Yangsan, Republic of Korea; 2 Department of Psychiatry and Biomedical Research Institute, Pusan National University Hospital, Busan, Republic of Korea; 3 Department of Public Health and Welfare, Finnish Institute for Health and Welfare (THL), Helsinki, Finland; 4 Department of Psychiatry, McGill University, Montreal, QC Canada; 5 Douglas Mental Health University Institute, Montreal, QC Canada

**Keywords:** Circadian regulation, Outcomes research

## Abstract

Exogenous melatonergic agents are widely used to treat insomnia and sleep disturbance. Several studies have shown that they might also modulate circadian rhythms. The purpose of this systematic review and meta-analysis was to summarize current knowledge about the effects of melatonin supplements and melatonin agonists on the sleep-wake cycle as well as on the circadian rhythm of melatonin in healthy participants and in patients with psychiatric disorders. The following electronic databases were searched: EMBASE, PubMed, Web of Science, CINAHL, and Cochrane Library. Of the 12,719 articles, we finally selected 30 studies including 1294 healthy participants and 8 studies including 687 patients with psychiatric disorders. Cochrane risk of bias tool was used to assess the risk of bias. Using meta-ANOVA, studies on healthy participants showed advancing effects of melatonergic supplements and agonists on sleep-wake cycle according to dosing time and dosage, despite the fact that the original individual melatonin rhythm was within a normal range (fixed effect model standardized mean difference *[95% Confidence Interval]* = −0.639[−0.968 to −0.310]). In a limited number of randomized controlled trials with psychiatric patients, the findings seemed similar to those with healthy participants, despite the psychiatric disorders and treatment related factors affecting circadian rhythms. Given the unmet clinical need for evidence-based treatments to correct circadian rhythms in psychiatric disorders, efficacy of melatonergic agents seen in healthy participants, and similarity of findings among psychiatric patients, large scale, well-designed randomized controlled trials are needed to test efficacy on circadian parameters in psychiatric disorders.

## Introduction

Melatonin is an endogenous hormone that is one of the key regulators of circadian rhythms in humans. The endogenous melatonin rhythm exhibits a close association with the endogenous component of the sleep propensity rhythm. Administration of exogenous melatonin is able: (i) to enhance sleepiness when the homeostatic drive to sleep is insufficient; (ii) to inhibit the drive for wakefulness emanating from the circadian pacemaker; and (iii) to induce phase shifts in the circadian clock such that the circadian phase of increased sleep propensity occurs at a new, desired time [1]. Melatonin secreted from the central nervous system is also one of the modulators of the hypothalamic–pituitary–adrenal axis [2].

Efficacy of melatonergic agents as exogenous regulators of sleep timing and circadian rhythm has been established in different conditions in which there is a problem with sleep-wake cycles. Melatonin has been shown to advance the timing of sleep in delayed sleep-wake phase disorder (DSWPD) [3]. In addition, the effects of melatonin including sleep-promoting, phase-shifting, and entrainment effects were investigated on jet lag symptoms [4, 5], adaptation of shift workers [6, 7], and entrainment in non-24-hour sleep-wake rhythm disorder (N24SWD) [8, 9]. Despite conflicting findings, several studies suggested potential effects on correcting and stabilizing circadian rhythm sleep-wake cycles [4, 5, 7–9]. Furthermore, there is some evidence for improving sleep quality in primary insomnia [10]. Data on melatonin receptor agonists are limited as compared to exogenous melatonin. Potential effects of tasimelteon have been reported in several studies with N24SWD, jet lag, and primary insomnia [11–15]. In addition, ramelteon has improved sleep quality and reduced latency to persistent sleep in primary insomnia, and corrected the timing of sleep in N24SWD [16, 17].

Patients with psychiatric disorders can show signs of disrupted circadian rhythms, particularly fragmentation and delayed phase, to the point where these characteristics have been considered as a core etiopathological factor [18]. Evidence for a disruption of circadian rhythm is robust especially in patients with bipolar disorders (BD) [19–22], including participants at high risk for BD, and is characterized by irregular rhythms, fragmentation and poor quality of sleep [20]. Patients with BD have also demonstrated a significantly lower peak of nocturnal melatonin levels (see a recent review [21]). Studies in patients with BD commonly report lower levels of overnight melatonin and delays in dim-light melatonin onset (DLMO) as compared to both patients with major depressive disorder (MDD) and healthy controls. However, one systematic review and meta-analysis with both BD and schizophrenia reported that effect sizes for sleep onset latency (SOL), total sleep time (TST), and wake after sleep onset (WASO) were in schizophrenia even higher than those in BD [23]. Half of patients with schizophrenia showed severe circadian misalignment, such as delayed/advanced sleep-wake phases or non-24-h sleep-wake patterns [24]. Furthermore, abnormalities in circadian rhythms in MDD or seasonal affective disorder have been reported [25]. Patients with MDD have showed eveningness chronotype, decreased amplitudes, and phase delays or advances of the circadian rhythms such as core body temperature and melatonin [26–28], while sleep disturbances and phase delays or advances of the circadian rhythms were observed in patients with seasonal affective disorder [29, 30]. While sleeping and eating related behaviors have shared regulatory systems [31], findings in eating disorders support internal dysregulation. Patients with night-eating syndrome had delayed and blunted rhythms of food intake, leptin, insulin and melatonin, as compared with those rhythms in healthy controls [32]. Additionally, there were significant differences in parameters measured with actigraphy, such as midline estimating statistic of rhythm and amplitude of rest-activity cycles between patients with binge eating disorder and healthy controls [33]. The late sleep phase was robustly associated with irregular eating pattern in a mixed sample of patients with eating disorders, also reflecting dysregulated sleep-wake cycles [34]. Patients with attention deficit hyperactivity disorder (ADHD) have shown increased nocturnal activity as well as daytime activity [35]. Especially, the absence of post-lunch dip was observed [36]. These findings might suggest that the circadian component was enhanced, while the homeostatic component was weakened [36]. Furthermore, in ADHD patients, DSWPD was frequently reported [37]. Finally, delayed and fragmented sleep was also detected in patients with borderline personality disorder [38]. Interestingly, abnormalities in the circadian rhythms appear very similar transdiagnostically across psychiatric disorders, hence raising issues of specificity and the possibility that the observed findings on circadian rhythms might be an epiphenomenon of the disorganization caused by any psychiatric disorder.

Several methods have been used in trials in order to capture the features of circadian rhythms. To this end, the three-oscillator model provides a framework for explaining some of these features, with the assessment of core body temperature, wake onset, and sleep onset [39]. The most common method in studies on sleep for the assessment of sleep stages from sleep onset to sleep offset has been polysomnography (PSG) [40–42]. Even though PSG is the current gold standard measurement for sleep, problems in longitudinal assessment have hindered its use for research on circadian rhythms [43]. For a PSG measurement, the need for several sensors, whether in a laboratory or ambulatory setting, limits its use in longitudinal studies and in patients with fragmented sleep [44]. Results of consecutive recordings by PSG could be affected by the first-night effect and night-to-night variability [45]. Furthermore, high costs limit large-scale research. Given these problems, PSG is not practical to measure sleep-wake cycles longitudinally [41]. Another method is using accelerometers and actigraphy to describe rest-activity cycles [21]. Yet not recording the sleep-wake cycles nor circadian rhythms directly, recent methodological developments have provided novel actigraphic parameters to better capture disrupted rhythms, including fragmentation index [46], midpoint of sleep, and sleep consolidation as well as their variability [47]. Meanwhile, one of accurate descriptors of the circadian rhythms is DLMO [48]. Utility of the gold standard using melatonin assays from blood, urine or saliva samples is limited for larger scale trials, since it necessitates repeated measurements and is sensitive to light exposure [21]. Overall, these proper circadian parameters have rarely been the focus of trials with melatonin. While an accumulating number of meta-analyses describe the efficacy of melatoninergic agents in specific target populations [16, 49–51], unfortunately, most of them focus on outcome measures other than circadian rhythm parameters.

In this systematic review, we wanted to summarize the current knowledge on randomized controlled trials (RCTs) with exogenous melatonin or a melatonin agonist to guide treatment of disrupted circadian and sleep-wake rhythms. The first aim was to synthesize accumulating evidence that melatoninergic agents in healthy participants can have a phase advancing effect on circadian rhythms and consolidate sleep. The second aim was to integrate the knowledge about the effects of melatonergic agents on rest-activity rhythms in psychiatric patients. The impact of exogenous melatonin in the psychiatric population could differ from that present in healthy participants due to factors which are related to the psychiatric disorder per se or other disorder-associated characteristics, most importantly, polypharmacy, behavioral factors, differences in external timers such as (lack of) work schedules, and obesity. While patients with distinct psychiatric disorders have commonly presented with delayed and disrupted rhythm sleep-wake disorders, melatonergic agents could be potentially optimal in treatment of these sleep problems. Thus, integrating knowledge about differences in the impact on circadian and sleep-wake rhythms between healthy participants and psychiatric patients is important to guide treatment of psychiatric patients but has not previously been done.

## Methods

We conducted a systematic review and, where possible, meta-analysis to investigate the effect of melatonergic agents in healthy participants and psychiatric patients. A systematic review and meta-analysis was undertaken according to the Preferred Reporting Items for Systematic reviews and Meta-Analyses guideline (Supplementary Table 1). The study protocol was registered at Open Science Framework (https://osf.io/hytxv/).

### Key question

The purpose of this review was to investigate the effects of melatonergic agents (e.g., exogenous melatonin, prolonged released melatonin, melatonin receptor agonists) on the parameters of melatonin rhythm and sleep-wake cycle as compared to placebo in healthy participants and psychiatric patients, and it had two specific aims as follows.

1. We investigated the effects of melatonergic agents on the sleep-wake cycle and circadian rhythms in healthy participants.
2. We explored the effects of melatonergic agents on the sleep-wake cycle and circadian rhythms in patients with any psychiatric disorder, a population with a high prevalence of abnormalities in circadian rhythms.

### Searching strategies

We searched for articles having “melatonergic agent-related keywords” AND “circadian rhythm-related keywords” AND “randomized controlled trial-related keywords” in their title and abstract. The search strategies comprised a combination of Medical Subject Headings (MeSH) or their equivalent (where available), keywords, truncations, and Boolean operators. The detailed search strategy is shown in the Supplementary Table 2. An electronic search was performed on EMBASE, PubMed, Web of Science, Cumulative Index to Nursing and Allied Health Literature (CINAHL), and Cochrane Library. All articles that were published from January 1980 to May 2020 were included.

### Study selection

Firstly, duplicated articles were removed electronically. Then, articles obtained by the search strategies were manually selected with the following methods. The inclusion criteria for articles were as follows: 1) a study of the influences of melatonergic agents on melatonin rhythm and sleep-wake cycle in healthy participants and in patients with psychiatric disorders, 2) RCT, 3) the age range from 18 to 65 years, and 4) data on internal rhythms, such as sleep-wake cycle and circadian melatonin rhythm. The exclusion criteria for articles were as follows: 1) contents irrelevant to the topic, 2) work in animals or cell models, 3) patients with dementia, neurological disorders, organic brain damages, autism, or intellectual disability, 4) patients with medical conditions known to affect circadian rhythms (e.g., cancer, surgery), 5) individuals undergoing pregnancy or lactation, 6) reports written by other languages except English, and 7) articles published before January 1, 1980.

**Fig. 1:**
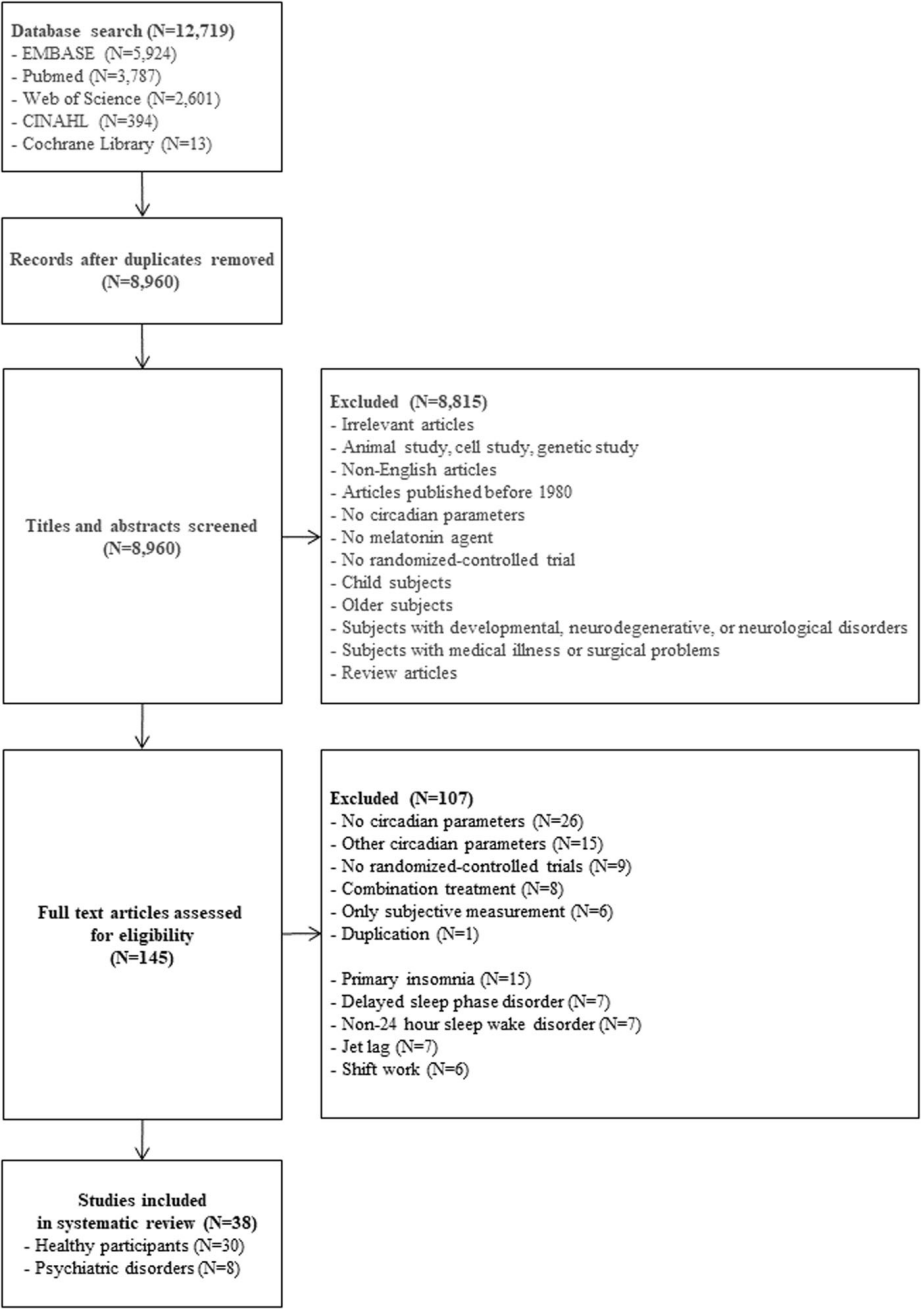
Flow chart of study selection. *N* Number.

The titles and abstracts of articles in a potential eligibility list were independently read by two authors (EM, OL) in order to evaluate the inclusion and exclusion criteria. Articles which met the exclusion criteria by both raters were removed from the potential eligible list. Full-texts of the remaining articles in the potential eligible list were independently read by two authors (EM, OL) in order to evaluate their eligibility. If there was any disagreement, we had a consensus meeting with a third review author (TP). References in eligible review articles were additionally evaluated by two authors (EM, OL) in order to find new articles (Fig. 1).

### Quality assessment

The quality assessments of RCTs were used by the Cochrane risk of bias tool [52]. Two authors (EM, OL) completed the quality assessment for each article independently. Disagreements were solved by the consensus meeting with a third review author (TP). The quality assessments of all included studies were described in Supplementary Table 3.

### Data extraction

Two authors (EM, OL) independently extracted data from the included studies (Tables 1, 2). If there was any disagreement, we discussed with a third member (TP) of the review team and finalized decisions. Structured forms were used for data extraction on the following contents: authors, publication year, country, study design, principal diagnosis of participants, agent used, sex, sample size, age range, drop-out, dosing time, duration of intervention, outcome measures, and main findings.

**Table 1:** Characteristics of included randomized controlled trials (*N* = 30) about the effects of melatonergic agents on circadian rhythm in healthy participants (*N* = 1294).

Authors(year)	Country	Study Design	Diagnosis	Melatonergic agent				Comparative agent				Dosing time	Intervention duration	Measurements				Main findings
				agent	Men/total *N*	age^a^	dropout	agent	Men/total *N*	age^a^	dropout			PSG	ACT	SLog	MEL	
**Exogenous melatonin (26 studies, total number of subjects** = **370)**																		
Arbon et al. [57]	UK	Cross-over	Healthy men and women	PR Melatonin 2 mg	12/16	58.8 (±2.9)	1/16	Placebo	12/16	58.8 (±2.9)	1/16	21:00	Single dose	+	-	-	+ (plasma,urine)	Reduced SWA No effects on PSG sleep parameters
Attenburrow et al. [59]	UK	Cross-over	Healthy male volunteers	Melatonin 0.5 mg	12/12	30 (21–37)	0/12	Placebo	12/12	30 (21–37)	0/12	17:00	1day, 7days	-	-	-	+ (plasma)	1-day melatonin: no effect on DLMO 7-day melatonin: advanced DLMO
Attenburrow et al. [58]	UK	Cross-over	Healthy middle-aged volunteers	Melatonin 0.3 mg, 1.0 mg	4/15	53.9 (41–67)	0/15	Placebo	4/15	53.9 (41–67)	0/15	2 h before bedtime	Single dose	+	-	-	-	Increased actual sleep time, sleep efficiency, shorter non-REM sleep and REM sleep latency
Burgess et al. [60]	UK	Cross-over Counter balanced	Healthy adults	Melatonin 0.5 mg, 3 mg	16/34	25.3 (±4.8)	0/34	Placebo	16/34	25.3 (±4.8)	0/34	various	Single dose	-	-	-	+ (saliva)	Calculating PRC on phase shift to melatonin
Cajochen et al. [61]^d^	Switzerland	Cross-over 8 h mini-constant routine protocol	Healthy young men	Melatonin 5 mg	8/8	Exp 1. 27 (4.0); Exp 2. 24.8 (3.5)	0/8	Placebo	8/8	Exp 1. 27 (±4.0) Exp 2. 24.8 (±3.5)	0/8	Exp 1. 18:00 Exp 2.13:00	Single dose	+ (EEG)	-	-	+ (saliva)	Significant correlation between melatonin levels and the timing of increased subjective sleepiness
Deacon et al. [62]	UK	Cross-over	Healthy males	Melatonin 5 mg	8/8	23–28	0/8	Placebo	8/8	23–28	0/8	17:00	Single dose	-	-	-	+ (saliva)	Advanced phase
Dijk et al. [63]	Switzerland	Cross-over Balanced	Healthy men	Melatonin 5 mg	8/8	22.4 (20–26)	0/8	Placebo	8/8	22.4 (20–26)	0/8	Immediately prior to a 4 h daytime sleep episode (13–17 h) after a partial sleep deprivation	Single dose	+ (EEG)	-	-	-	Enhanced power density in the 13.75-14.0 Hz and reduced activity in the 15.25-16.5 Hz
Dollins et al. [64]	US	Cross-over Latin square	Healthy male volunteers	Melatonin 0.1 mg, 0.3 mg, 1.0 mg, 10 mg	20/20	23.05 (±4.22)	0/20	Placebo	20/20	23.05 (±4.22)	0/20	11:45	Single dose	-	-	-	+ (serum)	Increased melatonin AUC Shorter sleep latency
Fisher et al. [65]	Germany	Cross-over	Blind individuals	Melatonin 5 mg	12/12	18–40	0/12	Placebo	12/12	18–40	0/12	1 h before bedtime	Single dose	+	-	-	+ (plasma)	Increased total sleep time and sleep efficiency
Holmes et al. [66]	Austria	Cross-over Counter balanced	Healthy young subjects	Melatonin 5 mg	7/12	20.3 (±0.6)	0/12	Placebo	7/12	20.3 (±0.6)	0/12	14:00	Single dose	+	-	-	-	Reduced sleep onset latency
Hughes et al. [67]	US	Cross-over Latin -square Counter balanced	Healthy young male subjects	Melatonin 1 mg, 10 mg, 40 mg	8/8	18–30	0/8	Placebo	8/8	18–30	0/8	10:00	Single dose	+	-	-	-	Shorter sleep onset latency, increased total sleep time and decreased wake after sleep onset
Kräuchi et al. [68]^c, d^	Switzerland	Cross-over Latin square Mini-constant routine protocol	Healthy male students	Melatonin 5 mg	8/8	27 (±4)	0/8	Placebo	8/8	27 (±4)	0/8	18:00	Single dose	-	-	-	+ (saliva)	Earlier DLMO
Matsumoto et al. [70]	Japan	Cross-over 8 h diurnal sleep protocol	Healthy male students	Melatonin 10 mg	6/6	23.7 (±1.3)	0/6	Placebo	8/8	23.7 (±1.3)	0/6	10:00		+	-	-	-	Increased total sleep time in diurnalsleep
Middleton et al. [71]	UK	Cross-over Partial temporal isolation under constant dimlight	Healthy males	Melatonin 5 mg	10/10	23.9 (±0.75)	1/10	Placebo	10/10	23.9 (±0.75)	1/10	20:00	15 days	-	-	+	+ (urine)	Phase advance (5/9), phase delay (2/9), and stabilization (2/9) of the sleep-wake cycle
Mishima et al. [72]^d^	Japan	Cross-over	Healthy male volunteers	Melatonin 3 mg, 9 mg	6/6	22.5 (±1.9)	0/16	Placebo	6/6	22.5 (±1.9)	0/16	9:30	Single dose	+(MSLT)	-	-	+ (serum)	Reduced sleep latency in MSLT and advanced endogenous melatonin rhythm
Nave et al. [73]^d^	Israel	Cross-over Latin-square	Young adults	Melatonin 3 mg, 6 mg	Unclear/12	24.6 (±2.7)	0/12	Placebo	Unclear/12	24.6 (±2.7)	0/12	16:00, 17:30	Single dose	+	+	+	-	Shortened sleep latency and increased total sleep time
Rajaratham et al. [74]	Austria	Cross-over Balanced	Healthy men	PR melatonin 1 mg.5 mg	8/8	24.4 (±4.4)	0/8	Placebo	8/8	24.4 (±4.4)	0/8	16:00	8 days	-	+	-	+ (plasma)	Advanced the timing of endogenous melatonin rhythm
Reid et al. [76]^d^	Australia	Parallel	Healthy young males	Melatonin 5 mg	16/16	20.3 (±2.4)^b^	0/16	Placebo	16/16	20.3 (±2.4)^b^	0/16	14:00	Single dose	+(MSLT)	-	-	-	Decreased sleep onset latency
Satoh et al. [79]	Japan	Cross-over	Healthy young male volunteers	Melatonin 0.5 mg, 3 mg, 9 mg	6/6	22.5 (19–24)	0/6	Placebo	6/6	22.5 (19–24)	0/6	9:30	Single dose	-	-	-	+ (serum)	Suppressed core body temperature
Seabra et al. [80]	Brazil	Parallel	Healthy male volunteers	Melatonin 10 mg	30/30	29 (±1)^b^	0/30	Placebo	10/10	29 (±1)^b^	0/10	1 h before sleep time22:00	28 days	+	-	-	-	Reduced stage 1 sleep
Stone et al. [81]	UK	Cross-over Latin-square	Healthy male volunteers	Melatonin 0.5 mg, 1 mg, 5, 10 mg	8/8	26.5 (21–31)	1/8	Placebo	8/8	26.5 (21–31)	1/8	23:30	Single dose	+	-	-	+ (saliva)	Increased total sleep time, sleep efficiency index and stage 2
Terlo et al. [82]	Israel	Cross-over	Healthy male volunteers	Melatonin 0.1 mg, 0.5 mg, and 1 mg	10/10	28 (±2)	0/10	Placebo	10/10	28 (±2)	0/10	16:00	Singledose	-	+	+	+ (urine)	No effects on sleep latency and efficiency Reduced wake time after sleep onset and delayed sleep offset time
Waldhauser et al. [83]^d^	Austria	Parallel	Healthy volunteers	Melatonin 80 mg	10/20	26.4 (±4.8)^b^	0/20	Placebo	10/20	26.4 (±4.8)^b^	0/20	21:00	Single dose	+(MSLT)	-	-	+ (serum)	Decreased sleep onset latency and increased sleep efficiency
Wirz-Justice et al. [84]^d^	Switzerland	Parallel Modified constant routine	Healthy young men	Melatonin 5 mg	9/9	23.6 (±2.8)	0/9	Placebo	9/9	23.6 (±2.8)	0/9	7:00	Single dose	-	+	-	+ (saliva)	Longer duration of higher-than-average temperature
Wright et al. [85]	UK	Cross-over	Healthy volunteers	Melatonin 2 mg	10/12 in spring 9/11 in autumn	22–46	0/12	Placebo	10/12 in spring 9/11 in autumn	22–46	0/11	17:00	1 month in spring 3 weeks in autumn	-	-	+	+	Increased sleep time and advanced the secretion of endogenous melatonin
Zhdanova et al. [86]	US	Cross-over Latin-Square	Healthy male volunteers	Melatonin 0.3 mg, 1.0 mg	6/6	26.5 (±1.3)	0/6	Placebo	6/6	26.5 (±1.3)	0/6	18:00, 20:00, 21:00	Single dose	+	-	-	-	Decreased sleep onset latency and latency to stage 2 sleep at any of the three time points
Agomelatine (1 study, total number of subjects = 8)																		
Kräuchi et al. [68]^c, d^	Switzerland	Cross-over Latin square Mini-constant routine protocol	Healthy male students	Agomelatine (S-20098) 5 mg, 100 mg	8/8	27 (±4)	0/8	Placebo	8/8	27 (±4)	0/8	18:00	Single dose	-	-	-	+ (saliva)	Earlier DLMO
Tasimelteon (1 study, total number of subjects = 450)																		
Rajaratnam et al. [74]	US	2 Parallel RCTs	Healthy individuals Transient insomnia	Phase II Tasimeleton 10 mg, 20 mg, 50 mg, 100 mg Phase III Tasimeleton 20 mg, 50 mg, 100 mg	Phase II 10mg (6/9) 20mg (4/8) 50mg (3/7) 100mg (3/7) Phase III 20mg (38/100) 50mg (44/102) 100mg (33/106)	Phase II 10 mg 31.8 (±7.4); 20 mg 32.5 (±9.6); 50 mg 27.4 (±6.2); 100 mg 30.4 (±9.5) Phase III 20 mg 30.8 (±8.4); 50 mg 31.0 (±8.5); 100 mg 31.2 (±8.2)	Phase II 1/31 Phase III 0/308	Placebo	Phase II 3/8 Phase III 35/103	Phase II 27.5 (6.7) Phase III 30.9 (7.3)	Phase II 1/8 Phase III 0/103	30 min before bedtime 5 h advance in the sleep-wake schedule	3 days	+	-	-	+ (plasma)	Phase II Improved sleep efficiency; Increased total sleep time; Shorter latency to sleep onset and persistent sleep; Advanced circadian rhythm shifting Phase III Improved sleep efficiency; Increased total sleep time; Shorter latency to sleep onset and persistent sleep; Decreased wake after sleep onset
Ramelteon (3 studies, total number of subjects = 464)																		
Markwald et al. [69]	US	Cross-over	Healthy female adults	Ramelteon 8 mg	9/14	23.2 (±4.2)	0/5	Placebo	9/14	23.2 (±4.2)	0/5	2 h prior to 4 h daytime sleep opportunity	Single dose	+	-	-	-	Reduced % wakefulness and wake after sleep onset; Increased TST, % stage 1, % stage 2
Richardson et al. [77]^d^	US	Parallel 5-hour shift advance	Healthy volunteers	Ramelteon 1 mg, 2 mg, 4 mg, 8 mg	1 mg (7/14) 2 mg (8/16) 4 mg (9/15) 8 mg (9/15)	1 mg 25.9 (±6.3) 2 mg 29.6 (±7.6) 4 mg 26.2 (±7.0) 8mg 26.1 (±5.7)	1 mg 0/14; 2 mg 0/16; 4 mg 0/15; 8 mg 1/15	Placebo	5/15	26.9 (±8.0)	0/15	30 min before bedtime 5 h advance in sleep-wakecycle	4 days	+	-	-	+ (saliva)	Advanced circadian phase (DLMoff): 1 mg,2 mg, 4 mg
Roth et al. [78]	US	Parallel	Healthy adults	Ramelteon 16 mg, 64 mg	16 mg (44/126); 64 mg (55/126)	16 mg 44.7 (±6.6) 64 mg 43.9 (±7.0)	16 mg 2/126; 64 mg 3/126	Placebo	47/123	44.0 (±7.1)	0/123	30 min before bedtime	Single dose	+	-	-	-	Shorter latency to persistent sleep; Longer total sleep time

*PR* prolonged release, *PSG* polysomnography, *ACT* actigraphy, *Slog* sleep log, *MEL* melatonin, *EEG* electroencephalogram, *SWA* slow-wave activity, *DLMO* dim light melatonin onset, *TST* total sleep time, *DLMoff* dim light melatonin offset, *REM* rapid eye movement, *PRC* phase response curve, *AUC* area under the curve, *MSLT* multiple sleep latency test

^a^Data on age were shown as mean ± standard deviation, or mean (age range) or are range.

^b^These data were reported in total group, not in separate groups.

^c^These studies are the same cohort.

^d^These studies used experimental designs such as napping, sleep deprivation, constant routine protocol or a phase shifting protocol.

**Table 2:** Characteristics of included randomized controlled trials (*N* = 8) about the effects of melatonergic agents on circadian rhythm in patients with psychiatric disorders (*N* = 687).

Authors (year)	Country	Study Design	Diagnosis	Melatonergic agent					Comparative agent				Dosing time	Intervention duration	Measurements				Main findings
				agent		Men/total *N*	age^a^	dropout	agent	Men/total *N*	age^a^	dropout			PSG	ACT	SLog	MEL	
Exogenous melatonin (3 studies, total number of subjects = 70)																			
Serfaty et al. [87]	UK	Parallel	DSM-IV MDE (UP or BP)	SR melatonin 6 mg	3/16	38.1 (11.6)	1/16	Placebo	1/17	42.0 (12.6)	1/16	Bedtime	4 weeks	-	+	+	-		No significant effects
Shamir et al. [88]	Israel	Cross-over	DSM-IV SPR	CR melatonin 2 mg	12/23	42 (5)	4/23	Placebo	12/23	42 (5)	4/23	2 h before desired bedtime	3 weeks	-	+	-	+ (urine)		Improved rest-derived sleep efficiency
Shamir et al. [89]	Israel	Cross-over	DSM-IV SPR	CR melatonin 2 mg	11/14	42.3 (13.1)	0/14	Placebo	11/14	42.3 (13.1)	0/14	2 h before desired bedtime	2 days	+	-	-	-		Enhanced first night effect: increase in REM sleep latency and the duration of wakefulness during sleep and decrease in sleep efficiency
Agomelatine (3 studies, total number of subjects = 461)																			
Kasper et al. [90]	Austria	Parallel	DSM-IV-TR MDD	Agomelatine 25 mg, 50 mg	41/154	43.3 (10.3)	21/154	Sertraline 50 mg, 100 mg	51/159	44.4 (10.2)	30/159	Evening	6 weeks	-	+	+	-		Higher RA and M10, lower L5, higher sleep efficiency, shorter sleep latency, and lower mean length of wake bouts
Quera-Salva et al. [91]	France	Parallel	DSM-IV MDD	Agomelatine 25 mg, 50 mg	23/71	41.3 (12.4)	23/71	Escitalopram 10 mg, 20 mg	26/67	41.4 (10.7)	23/67	Evening around 20:00	6 weeks	+	-	-	-		Shorter sleep latency and REM latency, lower number of sleep cycles
Saletu et al. [93]	Austria	Cross-over	DSM-IV MDD	Agomelatine 25 mg	Unclear/10	40.8 (10.4)	Unclear	Placebo	Unclear/10	40.8 (10.4)	Unclear	1 h before lights-off	Single dose	+	-	-	-		Improved sleep efficiency
Ramelteon (2 studies, total number of subjects = 156)																			
Fargason et al., [94]	US	Cross-over	DSM-IV Insomnia with ADHD	Ramelteon 8 mg	19/36	19–65	4/36	Placebo	19/36	19–65	4/36	30 min before desired sleep time 20:00–21:00	2 weeks	-	+	+	-		Phase advance (+): mean 45 min
Mishra et al. [95])	India	Parallel	DSM-5 SPR	Antipsychotics with ramelteon 8 mg add-on therapy	36/6;0 PG 15/30; NG 21/30	PG 38.6 (10.7) NG 34.9 (12.4)	0/300/30	Antipsychotics without ramelteon 8 mg add-on therapy	35/60 PG 21/30 NG 14/30	PG 34.0 (8.4); NG 37.9 (13.8)	0/30; 0/30	30 min before bedtime	4 weeks	-	-	-	+ (serum, urine)		Higher night-time melatonin level, AANAT, and urinary melatonin

*PSG* polysomnography; *ACT* actigraphy; *Slog* sleep log; *MEL* melatonin; *REM* rapid eye movement; *DSM* Diagnostic and Statistical Manual of Mental Disorders; *MDE* major depressive episode; *MDD* major depressive disorder; *UP* unipolar; *BP* bipolar; *SPR* schizophrenia; *ADHD* attention-deficit hyperactivity disorder; *SR* slow release; *CR* Controlled release; *PG* predominant positive symptom group; *NG* predominant negative symptom group; *RA* relative amplitude; *M10* the activity during a most active 10-h window; *L5* the activity during a least active 5-h window; *AANAT* arylalkylamine N-acetyltransferase

^a^Data on age were shown as mean ± standard deviation, or mean (age range), or age range.

^b^These data were reported in total group, not in sperate groups.

### Data synthesis

The meta-analysis of this study used the standardized mean differences (*SMD*) between comparative groups with 95% confidence interval (95% CI). When comparative data in multi-arm trials were used, the splitting method of shared groups was applied in order to maintain the characteristics of each arm [53]. Based on RCTs using exogenous melatonin in healthy participants, meta-ANOVA (analysis of variance) according to the dosing time and the dosage were performed. Additionally, studies using a melatonergic agonist as well as exogenous melatonin were compared using meta-ANOVA. The heterogeneity of the results of meta-analysis was evaluated with an *I*^*2*^ statistics (a low level of <25%; a moderate level of 25–50%; a high level of >50%) [54]. When indicating significant heterogeneity based on the *p* value of heterogeneity test and *I*^*2*^ statistics (>50%: a high level of heterogeneity), a random effect model was adopted [54–56]. Sensitivity analysis was performed by omitting one individual study at a time. Potential publication bias was evaluated with the linear regression test of funnel plots. A two tailed *p-*value less than 0.05 was considered as of a statistical significance. All the statistical analyses were performed by using R version 4.0.5 with the “meta” package for meta-analysis.

## Results

### Study selection

The original search strategy retrieved 12,719 studies, after which 3759 duplicate studies were excluded (Fig. 1). Next, after screening the titles and abstracts according to the eligibility criteria by two independent reviewers (EM, OL), 8815 studies were excluded. Two independent reviewers (EM, OL) further evaluated a total of 145 studies by reading the full-text articles, and 65 of them were excluded. Finally, we identified 80 eligible studies and excluded 42 articles which were not related to the topic. Thus, in this systematic review, 30 studies on healthy participants (*N* = 1294) and 8 studies on patients (*N* = 687) with psychiatric disorders were addressed.

### Current evidence on effects of melatonergic agents in healthy participants

The 30 RCTs on effects of melatonergic agents in healthy participants were selected as shown in Table 1 [57–86]. These studies administered exogenous melatonin (26/30, 86.7%), agomelatine (1/30, 3.3%), ramelteon (3/30, 10%), and tasimelteon (1/30, 3.3%). All the RCTs including exogenous melatonin, agomelatine, ramelteon, and tasimelteon reported significant effects on at least one of parameters on sleep-wake cycle or circadian melatonin rhythm. However, the effect sizes of melatonergic agents varied according to dosage and dosing time, respectively.

### The results of meta-analysis on RCTs using exogenous melatonin in healthy participants

**Fig. 2:**
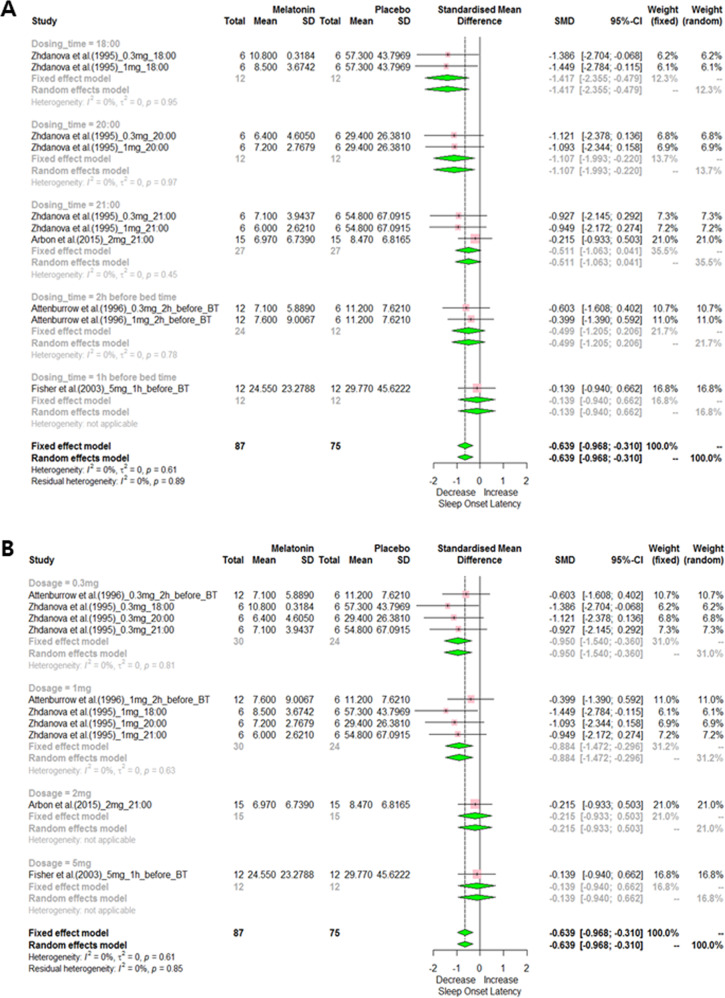
The synthesized standardized mean difference (SMD) of 10 comparative datasets. The pooled SMD in healthy subjects showed that exogenous melatonin significantly decreased SOL compared to placebo. Administration of exogenous melatonin at 18:00 and 20:00 significantly decreased SOL (**A**). Low dosages significantly shortened SOL (**B**). BT bedtime, SD standard deviation.

The effects of exogenous melatonin on sleep parameters in healthy participants were compared according to the dosing time and the dosage using meta-ANOVA (Fig. 2A, B). In comparing efficacy according to dosing time, administration of exogenous melatonin at 18:00 h and 20:00 h significantly decreased SOL (Fig. 2A). According to dosage, relatively low dosages such as 0.3 mg and 1 mg significantly shortened SOL, while relatively high dosages such as 2 mg and 5 mg did not show a significant effect on SOL (Fig. 2B). Using exogenous melatonin 5 mg at an hour before the bedtime also significantly increased sleep efficiency (SE) (Supplementary Fig. S1A, B). There were no significant effects of exogenous melatonin on TST (Supplementary Fig. S2A, B) or WASO (Supplementary Fig. S3A, B), with the exception of one study using exogenous melatonin 5 mg at 1-h before bedtime which significantly increased TST (Supplementary Fig. S2A, B). Sensitivity analysis showed that the results of meta-analysis were not changed after removing any one of the analyzed data. When classifying included studies according to heterogeneity, the number of included studies was limited to evaluate small study effects or potential publication bias.

### Comparison of RCTs using melatonergic agents on sleep parameters in healthy participants

**Fig. 3:**
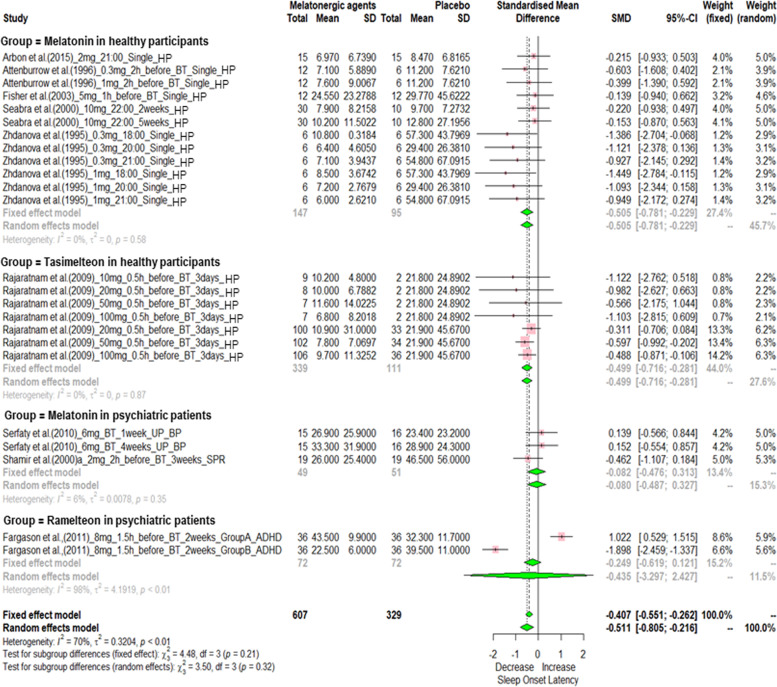
Meta-ANOVA on efficacy of exogenous melatonin and melatonergic agents on sleep onset latency (SOL) in healthy participants and psychiatric patients. The standardized mean differences (SMD) of 24 comparative datasets were synthesized. The pooled SMD in healthy participants showed that exogenous melatonin and tasimelteon significantly decreased SOL compared to placebo. The pooled SMD of exogenous melatonin and ramelteon in psychiatric patients did not show change of SOL compared to placebo. HP healthy participants, UP unipolar, BP bipolar, ADHD attention deficit hyperactivity disorder, SPR schizophrenia, BT bedtime, SD standard deviation.

In order to compare the effect size of these studies according to the melatonergic agent, meta-ANOVA was performed for RCTs which reported the same sleep parameters. In analysis of meta-ANOVA, exogenous melatonin and tasimelteon significantly shortened SOL (Fig. 3) and improved SE (Supplementary Fig. S4) in healthy participants. The effect sizes on SOL with exogenous melatonin and tasimelteon were similar (SMD: melatonin -0.505 vs. tasimelteon −0.499; see Fig. 3). Meanwhile, the effect size of tasimelteon on SE was relatively large as compared to that of exogenous melatonin (SMD, melatonin 0.332 vs tasimelteon 0.528) (Supplementary Fig. S4). In addition, ramelteon and tasimelteon significantly increased TST in healthy participants, with no effect from exogenous melatonin on TST (Supplementary Fig. S5). The effect size of tasimelteon was relatively large as compared to that of ramelteon (SMD: ramelteon 0.329 vs tasimelteon 0.526; see Supplementary Fig. S5). Tasimelteon significantly decreased WASO (SMD: tasimelteon −0.372), with no effect from exogenous melatonin or ramelteon on WASO (Supplementary Fig. S6). Sensitivity analysis showed unchanged results after removing any one of the analyzed data. The limited number of included studies did not allow us to evaluate small study effects or potential publication bias.

### Current evidence on effects of melatonergic agents in patients with psychiatric disorders

A total of eight RCTs using melatonergic agents examined the effects on parameters of sleep-wake cycle or circadian melatonin rhythms in patients with psychiatric disorders (Supplementary Table 4). Given the methodological differences and limited number of reports, we first describe results from individual studies. Three RCTs used exogenous melatonin in psychiatric patients [87–89]. In one RCT with 19 schizophrenia patients, controlled-release melatonin 2 mg showed significant improvement in SE as compared to placebo [88]. Another RCT examined the first-night effect in PSG under the unfamiliar sleeping environment in 14 patients with schizophrenia [89]. The first night effect indicates sleep disturbances such as a decreased sleep efficiency, an increasedSOL, and increased awakening due to vigilance in an unfamiliar sleeping condition. This study showed that controlled-release melatonin 2 mg worsened sleep states, such as a longer REM sleep latency, a lower SE, and an increased WASO in patients with schizophrenia, as compared to placebo. In contrast, a third RCT with two parallel groups including 33 patients with a major depressive episode (MDD or BD) reported no significant impact on sleep parameters as measured with wrist actigraphy between slow-release melatonin 6 mg and placebo [87].

Three RCTs described the effects of agomelatine in psychiatric patients [90–92]. One RCT described the effects on sleep parameters from actigraphy in 313 patients with MDD, divided to groups with agomelatine or sertraline. They reported that patients treated with agomelatine 25 mg and 50 mg for 6 weeks showed a higher M10 (the activity during the most active 10-h period per day), a lower L5 (the activity during the least active 5-h period per day), a higher relative amplitude, a shorter SOL, a higher SE, a lower mean length of wake bouts for each night than patients on sertraline treatment [90]. Another RCT comparing the effects of agomelatine and escitalopram on sleep parameters as assessed with PSG in 138 patients with MDD observed that agomelatine 25 mg and 50 mg for 6 weeks significantly reduced sleep latency as well as REM latency, and decreased the number of sleep cycles as compared to escitalopram 10 mg or 20 mg [91]. In another RCT with ten patients having MDD, agomelatine 25 mg also shortened SOL, improved SE, and increased TST [93].

There were two RCTs on the effects of ramelteon in psychiatric patients [94, 95]. One RCT in 120 patients with schizophrenia on antipsychotics reported that ramelteon 8 mg as add-on medication significantly increased the night-time melatonin, serum arylalkylamine N-acetyltransferase (AANAT), and urinary 6MTas levels as compared to only antipsychotics without the add-on ramelteon [95]. Increase of serum AANAT concentration known as melatonin rhythm-generating enzyme [96] was consistent with melatonin secretion. Another RCT in 36 patients with insomnia and adult ADHD showed that patients after two weeks of treatment with ramelteon 8 mg had a significantly earlier sleep midpoint as measured with actigraphy than those on placebo [94].

### The results of meta-analysis on RCTs using exogenous melatonin and melatonergic agents in psychiatric patients

When synthesizing findings in psychiatric patients, there were no significant effects of exogenous melatonin and ramelteon on SOL (Fig. 3), SE (Supplementary Fig. S4), TST (Supplementary Fig. S5), and WASO (Supplementary Fig. S6). Due to the small number and methodological differences of studies, the meta-analyses according to dosing and timing were not performed. Even though some studies showed significant effects of agomelatine on sleep parameters, these studies were not included in the meta-analysis due to use of comparative antidepressants instead of placebo or limited information (only poster abstract available). The main findings of these studies not included in meta-ANOVA were summarized in Supplementary Table 4. Of the two studies using exogenous melatonin included in meta-analysis, one study used SR melatonin 6 mg at bedtime, and the other study used CR melatonin 2 mg at 2 h before desired bedtime. These dosages and dosing time of exogenous melatonin were similar to those that did not show the significant findings in healthy participants (Fig. 2). While large-scale studies in healthy participants showed significant findings on sleep-wake parameters, studies administrating ramelteon in psychiatric patients showed a similar finding with a small sample size (each group’s *N* = 36, cross-over design).

## Discussion

This systematic review summarized the effects of melatonergic agents for correcting disrupted sleep-wake and circadian rhythms in healthy and psychiatric participants. Trials on healthy participants demonstrated that specific melatonergic supplements and agonists advanced the phase of sleep-wake and circadian rhythms that were originally within the normal range. The meta-analysis of studies with sleep-wake cycle parameters showed that the exogenous melatonin and melatonergic agonists significantly advanced the phase of circadian melatonin rhythm [59, 60, 62, 68, 74, 77, 85]. Given an advancing effect of melatonin on circadian parameters [68, 74, 94], shortening sleep latency might be caused by a chronobiotic effect such as a phase advance. Alternatively, these effects of exogenous melatonin and melatonergic agents on sleep parameters could be mediated by a hypnotic effect and/or sleep consolidation [16, 49, 97]. Particularly, the co-occurrence of a decreased SOL and an advanced DLMO in the delayed sleep phase syndrome suggest the possibility that shortening of sleep latency might be related to the advance of circadian rhythms such as measured with DLMO [3].

In psychiatric populations, the prevalence of phase delay and disrupted circadian rhythms is high. Despite the need for evidence-based treatments for phase delay and disrupted circadian rhythms specifically in psychiatric populations, our main finding for this population was that trials with melatonergic agents and with a focus on circadian parameters are very limited and methodologically too variable for conclusions. Interestingly, the effect of melatonergic agents seemed similar to that of healthy participants [88–91, 94]. Studies using agomelatine in psychiatric patients showed an improving effect on sleep-wake parameters [90, 91, 93], similar to the results in healthy participants.

Meanwhile, studies with psychiatric patients reported stabilizing effects on circadian and sleep-wake rhythms, such as sleep-wake cycle or melatonin rhythm [88, 90, 91, 93–95], similar to the results in healthy participants. Among the three trials in patients with schizophrenia, one RCT with exogenous melatonin for three weeks improved sleep-wake cycle [88], and another RCT with add-on ramelteon medication enhanced circadian melatonin rhythm [95]. Among the four trials in patients with MDD, one RCT using slow-release melatonin 6 mg with major depressive episode including bipolar depression did not show any significant finding on sleep-wake parameters as measured with actigraphy and sleep log [87]. While three RCTs including two large MDD cohorts showed a consistent positive effect of agomelatine on sleep-wake cycle, two studies compared agomelatine to selective serotonin reuptake inhibitors (SSRIs) instead of placebo. When considering the evidence that SSRI can change sleep pattern [98], these findings should be cautiously interpreted. A trial with ADHD also reported that ramelteon advanced the timing of sleep-wake cycle [94]. For other potential agents to correct circadian rhythms, in our previous review, we found that antipsychotics actually might be more harmful and rhythm disrupting [99]. The negative impact of selective serotonergic agents has been recognized earlier [98], and the use of benzodiazepines has contraindications [100]. We conclude that melatonergic agents have shown potential efficacy in and are the most promising agents for correcting disrupted sleep-wake and circadian rhythms. Given the clinical need for evidence-based treatments to correct circadian rhythms, further trials with proper inclusion criteria and outcome measures for circadian rhythms among psychiatric patients are warranted. Limited data provides some evidence that melatonin might be less efficient on sleep-wake parameters than ramelteon or agomelatine in this patient population. Trials measuring the effects of especially agomelatine and ramelteon in different psychiatric cohorts on sleep phasing and consolidation are warranted.

Several studies have suggested that melatonergic agents can be effective as antidepressant or antimanic agent [101–103]. Given that disrupted circadian rhythms are related to mood symptoms [104], the antidepressant or antimanic effect of melatonergic agents may be at least partly be mediated through correcting disrupted circadian rhythms [105, 106]. According to the findings of our meta-analysis, the effect of melatonergic compounds was dependent on the dosing time and dose. Based on the phase–response curve and the results of this meta-analysis, the dosing time around 3 h before DLMO from 18:00 h to 20:00 h is likely to be the best dosing time to advance phasing., and the optimal dose was small. With this timing, the effect sizes of sleep-wake parameters (SOL) were large [107–109]. These results were consistent with earlier results which established the phase–response curve for shifting the phase of melatonin rhythm [48, 110]. A small dosage of melatonin showed a bigger effect size of advancing SOL than a high dosage. A high dosage of melatonin, however, was more effective to increase SE and/or TST. Considering the differential effects of dosage according to sleep-wake parameters, the clinician has to select the proper timing and dosage depending on treatment target such as reducing SOL or increasing TST. A well-designed study that can confirm the dose–response curve for specific sleep-wake parameters is needed in the future. If confirmed, these results could guide personalized treatment.

Chronobiological and chronotherapeutic knowledge should inform methods of future trials. Optimal parameters to objectively describe the phase shifting of circadian rhythms and poor sleep consolidation are important, and they were rarely used in trials, especially for psychiatric patients. Typically, the individual rhythms were not evaluated on pre-trial basis, while the dosing time was predetermined and thus not matched with the individual rhythm. In other words, the efficacy of melatonergic agents according to the predetermined dosing time could be different on each person and the mean effect of melatonergic agents is likely to be dampened. The melatonin level could be affected by various factors such as sex, age, current mood, seasons, medications, and exposure to light [111–113] and should be better controlled for in future trials. The study design, including the compound, timing and dose, inclusion criteria, and outcome, should be suitable for the target circadian outcome. It seems that transdiagnostic psychiatric cohorts with uniform circadian inclusion criteria would work, which would make it easier to recruit larger cohorts providing clinically relevant data [114]. In these carefully characterized cohorts, more knowledge about an optimal dosing time as compared to the current individual phasing, period and target timing can be gained.

### Limitations

Firstly, despite the reasonable number of original studies on healthy participants, the methodological variability reduced the number of comparable studies for the meta analysis. Nevertheless, our main findings concern results from the meta-analysis that showed a low heterogeneity based on *I*^2^ within the included articles and a low risk of publication bias. Secondly, of the number of studies in psychiatric patients was limited, and additionally, heterogeneity of clinical diagnosis, and heterogeneity of parameters of circadian rhythms limited conclusions for this population. We found no trials in anxiety disorders, eating disorders, personality disorders, or specifically in BD. Most of the studies on the effects of melatonergic agents stick to a classic clinical study design, inclusion by diagnostic category, and mood as the main outcome. We focused on studies with objective circadian parameters and thus, excluded a number of studies with psychometric measures of insomnia or mood as outcomes.

## Conclusion

In healthy participants, most of studies with exogeneous melatonin showed an advancing effect of circadian rhythm. Interestingly, for phase advancement an earlier time and smaller dose, but for sleep quality/consolidation a larger and later dose seemed appropriate. Exogenous melatonin and melatonergic receptor agonists could be effective in correcting disrupted circadian and sleep-wake rhythms. Large scale, well-designed randomized controlled trials in psychiatric patients are warranted.

## Supplementary information

Supplementary Table 1

Supplementary Table 2

Suppl Tables 3 - 4 & Figures 1- 6
