# Supplementary material for: Melatonergic agents influence the sleep-wake and circadian rhythms in healthy and psychiatric participants: a systematic review and meta-analysis of randomized controlled trials
Source: Neuropsychopharmacology. 2022 Feb 4;47(8):1523–36. doi: 10.1038/s41386-022-01278-5 (PMC9206011; doi:10.1038/s41386-022-01278-5)
Supplement: Supplementary file 3 — Suppl Tables 3 - 4 & Figures 1- 6 [file 41386_2022_1278_MOESM3_ESM.pdf]

Supplementary Table 3. Summary of quality assessment (risk of bias) for included 36 randomized controlled trials

Authors (year)	Random sequence generation (selection bias)	Allocation concealment (selection bias)	Blinding of participants and personnel (performance bias)	Blinding of outcome assessment (detection bias)	Incomplete outcome data (attrition bias)	Selective reporting (reporting bias)	Other bias	Total Risk of Bias
**Arbon et al. (2015)**	**L**	**L**	**L**	**L**	**L**	**L**	**L**	**0**
**Attenburrow et al. (1995)**	**U**	**U**	**U**	**L**	**L**	**L**	**H**	**5**
**Attenburrow et al. (1996)**	**U**	**U**	**U**	**L**	**L**	**L**	**U**	**4**
**Burgess et al. (2010)**	**U**	**U**	**U**	**L**	**L**	**L**	**U**	**3**
**Cajochen et al. (1996)**	**U**	**U**	**U**	**L**	**L**	**L**	**U**	**3**
**Deacon et al. (1994)**	**U**	**U**	**U**	**L**	**L**	**L**	**U**	**4**
**Dijk et al. (1995)**	**U**	**U**	**U**	**L**	**L**	**L**	**U**	**4**
**Dollins et al. (1994)**	**U**	**L**	**L**	**L**	**L**	**L**	**U**	**2**
**Fargason et al., (2011)**	**U**	**L**	**L**	**L**	**L**	**L**	**H**	**3**
**Fisher et al. (2003)**	**U**	**U**	**U**	**L**	**L**	**L**	**U**	**4**
**Holmes et al. (2002)**	**U**	**L**	**U**	**L**	**L**	**L**	**U**	**3**
**Hughes et al. (1997)**	**U**	**L**	**U**	**L**	**L**	**L**	**U**	**3**
**Kasper et al. (2010)**	**U**	**L**	**L**	**L**	**L**	**L**	**L**	**1**
**Kräuchi et al. (1997)**	**U**	**U**	**L**	**L**	**L**	**L**	**U**	**3**
**Markwald et al. (2010)**	**L**	**L**	**L**	**L**	**L**	**L**	**H**	**2**
**Matsumoto et al. (1999)**	**U**	**U**	**U**	**L**	**L**	**L**	**U**	**4**
**Middleton et al. (1997)**	**U**	**U**	**L**	**L**	**L**	**L**	**U**	**3**
**Mishima et al. (1997)**	**U**	**L**	**L**	**L**	**L**	**L**	**U**	**2**
**Mishra et al. (2020)**	**L**	**L**	**U**	**L**	**L**	**L**	**L**	**1**
**Nave et al. (1995)**	**U**	**U**	**U**	**L**	**L**	**L**	**U**	**4**
**Quera-Salva et al. (2011)**	**U**	**L**	**L**	**L**	**L**	**L**	**H**	**3**
**Rajaratham et al. (2003)**	**U**	**L**	**U**	**L**	**L**	**L**	**U**	**3**
**Rajaratnam et al. (2009)**	**L**	**L**	**L**	**L**	**L**	**L**	**L**	**0**
**Reid et al. (1996)**	**U**	**U**	**U**	**L**	**L**	**L**	**U**	**4**
**Richardson et al. (2008)**	**U**	**U**	**U**	**L**	**L**	**L**	**H**	**5**
**Roth et al. (2005)**	**U**	**U**	**U**	**L**	**L**	**L**	**H**	**5**
**Saletu et al. (2012)**	**U**	**U**	**U**	**L**	**U**	**U**	**H**	**7**
**Satoh et al. (2001)**	**U**	**L**	**L**	**L**	**L**	**L**	**U**	**2**
**Seabra et al. (2000)**	**U**	**L**	**U**	**L**	**L**	**L**	**U**	**3**
**Serfaty et al. (2010)**	**L**	**L**	**L**	**L**	**L**	**L**	**U**	**1**
**Shamir et al. (2000a)**	**U**	**L**	**L**	**L**	**L**	**L**	**H**	**3**
**Shamir et al. (2000b)**	**U**	**L**	**U**	**L**	**L**	**L**	**U**	**3**
**Stone et al. (2000)**	**L**	**L**	**U**	**L**	**L**	**L**	**U**	**2**
**Terlo et al. (1997)**	**U**	**L**	**U**	**L**	**L**	**L**	**U**	**3**
**Waldhauser et al. (1990)**	**U**	**L**	**U**	**L**	**L**	**L**	**U**	**3**
**Wirz-Justice et al. (2002)**	**U**	**U**	**U**	**L**	**L**	**L**	**U**	**4**
**Wright et al. (1986)**	**U**	**L**	**U**	**L**	**L**	**L**	**U**	**3**
**Zhdanova et al. (1995)**	**U**	**L**	**U**	**L**	**L**	**L**	**U**	**3**

Low risk of bias (L) was scored as 0 point, high risk of bias (H) as 2 point, unclear risk of bias (U) as 1 point, respectively. Total risk of bias was evaluated based on total summation score of each item as following criteria: ≦ 6 total score was scored as low risk of bias > 6 total score was scored as high risk of bias.

Supplementary Table 4. Effects of exogenous melatonin and melatonergic agents in patients with psychiatric disorders

Study	Diagnosis	Agents	Index to agents	Principal parameters					Remarks
				SOL	SE	TST	WASO	Mel	
**Exogenous melatonin**									
Serfaty et al. (2010)	DSM-IV MDE (UP or BP)	SR melatonin 6mg	Bedtime	↔	↔	↔	↔	NA	Melatonin group showed non-significantly higher WASO and lower SE at baseline.
Shamir et al. (2000a)	DSM-IV SPR	CR melatonin 2mg	2 hours before desired bedtime	↔	↑	↔	↔	6-SMT	6-SMT only measured to evaluate the melatonin levels at baseline.
Sharmir et al. (2000b)	DSM-IV SPR	CR melatonin 2mg	2 hours before desired bedtime	NA	NA	NA	NA	NA	Designed to describe the first-night effect, not to detect the effect of melatonin on sleep. First-night effects were stronger in melatonin group than placebo group.
**Agomelatine**									
Kasper et al. (2010)	DSM-IV-TR MDD	Agomelatine 25mg, 50mg vs. Sertraline 50mg, 100mg	Evening	↓	↑	NA	NA	NA	The effects of agomelatine on sleep were compared to sertraline group, not placebo.
Quera-Slava et al. (2011)	DSM-IV MDD	Agomelatine 25mg, 50mg vs. Escitalopram 10mg, 20mg	0~60 min before bedtime	↓	↔	↔	↔	NA	The effects of agomelatine on sleep were compared to escitalopram group, not placebo.
Saletu et al. (2012)	DSM-IV MDD	Agomelatine 25mg vs. Placebo	1 hour before lights-off	↓	↑	↑	↔	NA	A poster only, methods and results were not provided in detail.
**Ramelteon**									
Fargason et al. (2011)	DSM-IV Insomnia with ADHD	Ramelteon 8mg vs. Placebo	30 min before desired sleep time 20:00-21:00	↔ (↓ in group B)	↔	↔	NA	NA	Trials of two groups (A: ramelteon to placebo, B: placebo to ramelteon) were performed. Although the total group of participants showed no effects of ramelteon on sleep, Group B showed reduced sleep latency. However, the phase of mid-sleep time calculated by sleep start and end times was significantly advanced by ramelteon through two-way ANOVA analysis (Medication time X Drug)
Mishra et al. (2020)	DSM-5 SPR	Antipsychotics with ramelteon 8mg add-on therapy vs. Antipsychotics without ramelteon 8mg add-on therapy	30 min before bedtime	NA	NA	NA	NA	Night-time serum Mel↑ Urine Mel↑	Comparison between positive and negative psychotic symptom groups

Abbreviations: SOL, sleep onset latency; SE, sleep efficiency; TST, total sleep time; WASO, wake after sleep onset; Mel, melatonin; SR, slow release; CR, controlled release; 6-SMT, 6-sulfatoxymelatonin; NA, not applicable; ANOVA, analysis of variance

A

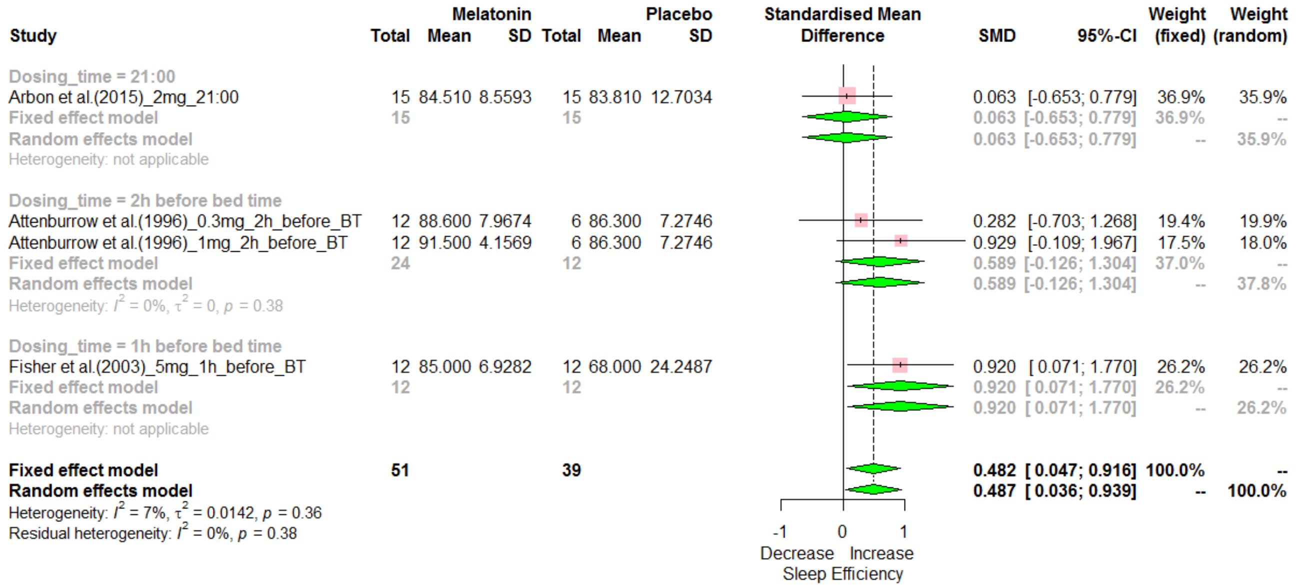

B

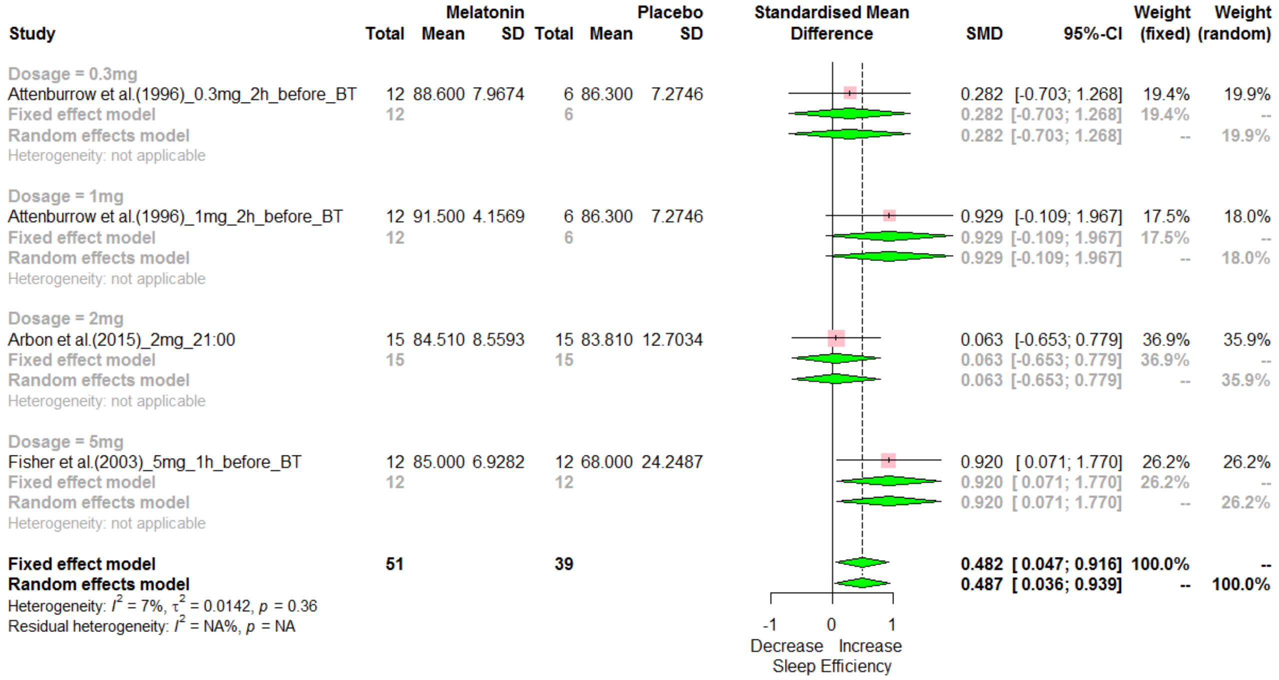

Supplementary Figure 1. Meta-ANOVA on efficacy of exogenous melatonin on sleep efficiency (SE) in healthy participants according to dosing time (A) and dosage (B). The standardized mean difference (SMD) of 4 comparative datasets were synthesized. The pooled SMD in healthy participants showed that exogenous melatonin significantly increase SE compared to placebo (Heterogeneity I^2=7%, τ^2=0.0142, p=0.36, fixed effect model *SMD [95% CI]* = 0.482[0.047 ~ 0.916]). Melatonin administered at an hour before the bed time had significant increase on SE (Supplementary Figure S2A) Melatonin 5mg significantly increase SE (Supplementary Figure S2B)

A

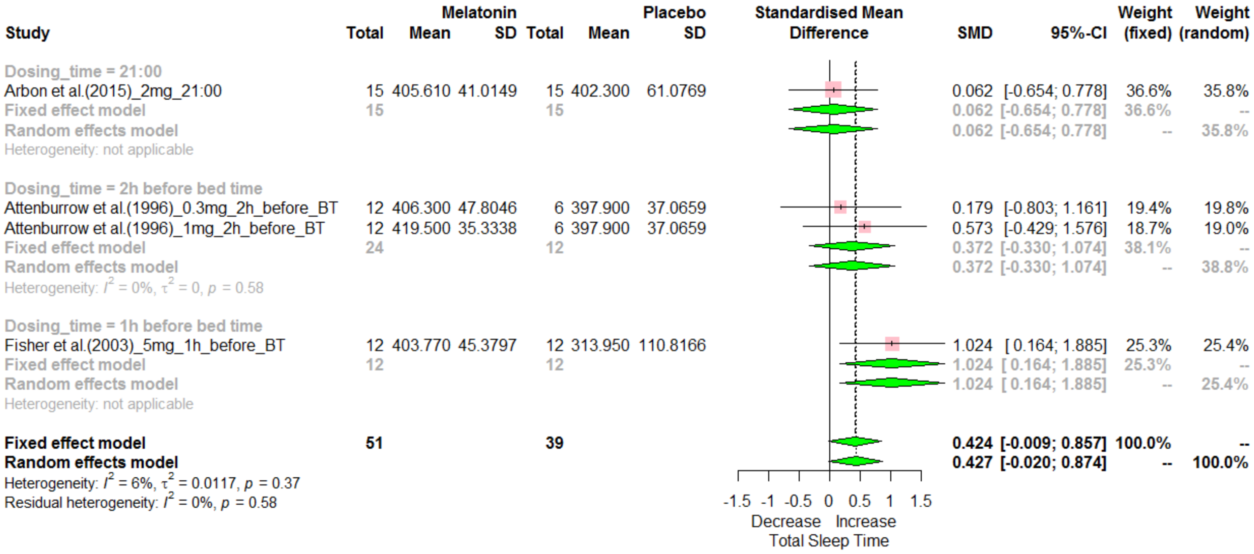

B

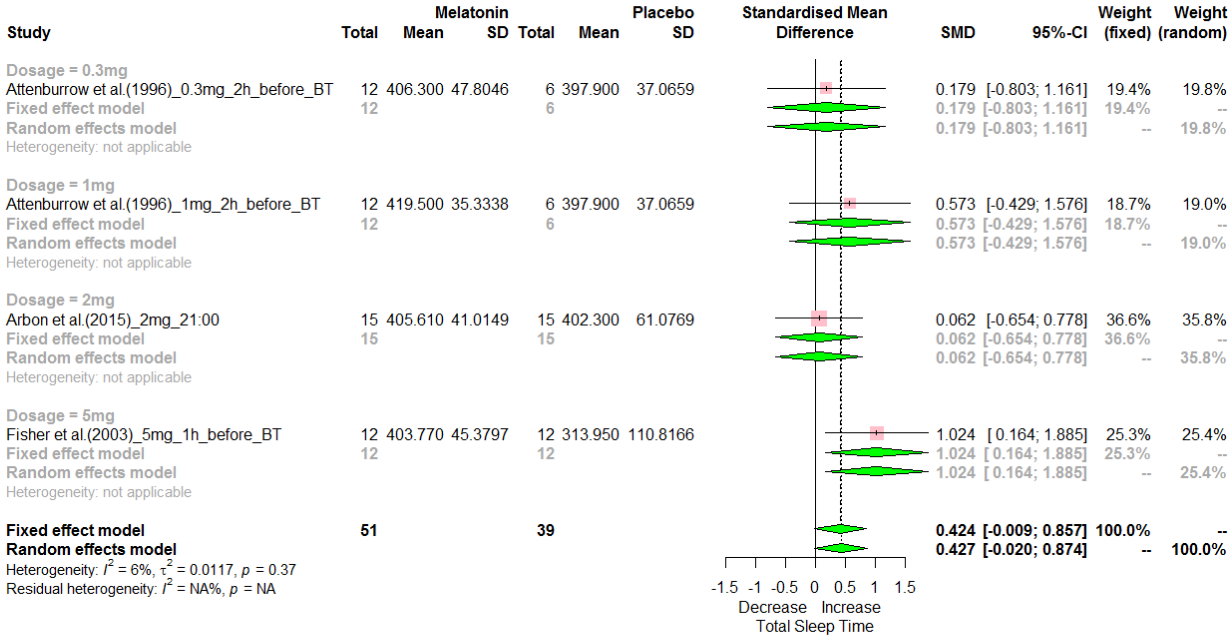

Supplementary Figure 2. Meta-ANOVA on efficacy of exogenous melatonin on total sleep time (TST) in healthy participants according to dosing time (A) and dosage (B). The standardized mean difference (SMD) of 4 comparative datasets were synthesized. The pooled SMD in healthy participants showed that exogenous melatonin did not significantly increase TST compared to placebo (Heterogeneity I^2=6%, τ^2=0.0117, *p*=0.0117, fixed effect model *SMD [95% CI]* = 0.424[-0.009 ~ 0.857]). However, melatonin administered at 1-hour before bed time had significant increase on TST (Supplementary Figure S3A) Melatonin 5mg significantly increase SE (Supplementary Figure S3B).

A

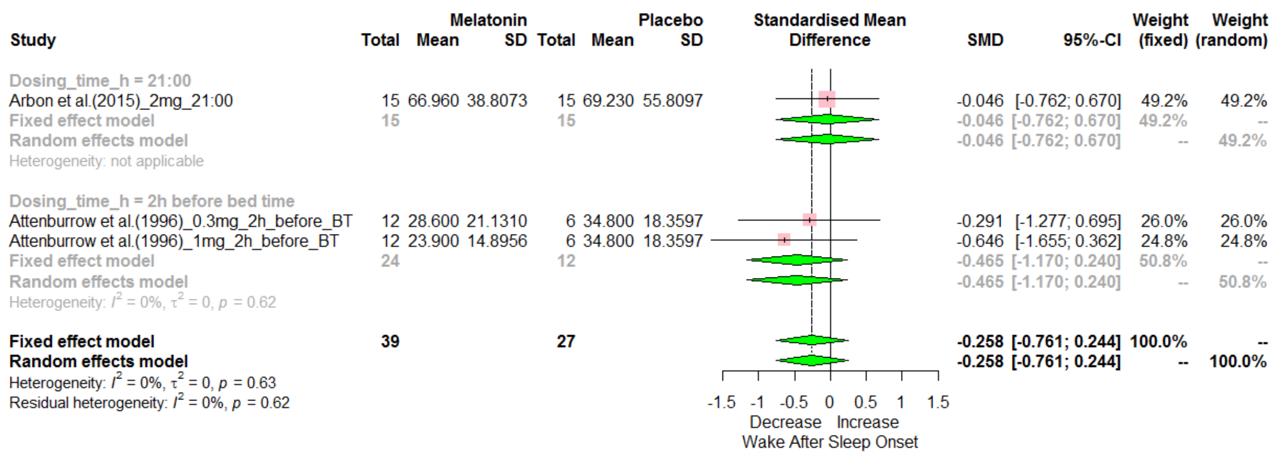

B

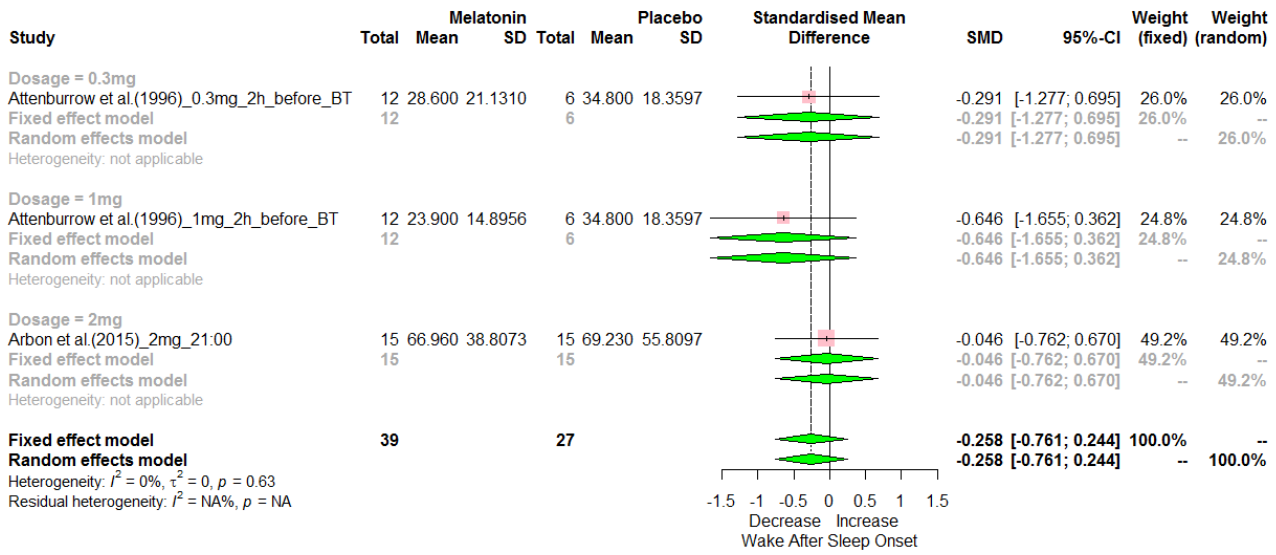

Supplementary Figure 3. Meta-ANOVA on efficacy of exogenous melatonin on wake after sleep onset (WASO) in healthy participants according to dosing time (A) and dosage (B). The standardized mean difference (SMD) of 3 comparative datasets were synthesized. The pooled SMD in healthy participants showed that exogenous melatonin did not significantly increase WASO compared to placebo (Heterogeneity I^2=0%, τ^2=0, p=0.63, fixed effect model *SMD [95% CI]* = -0.258[-0.761 ~ 0.244]).

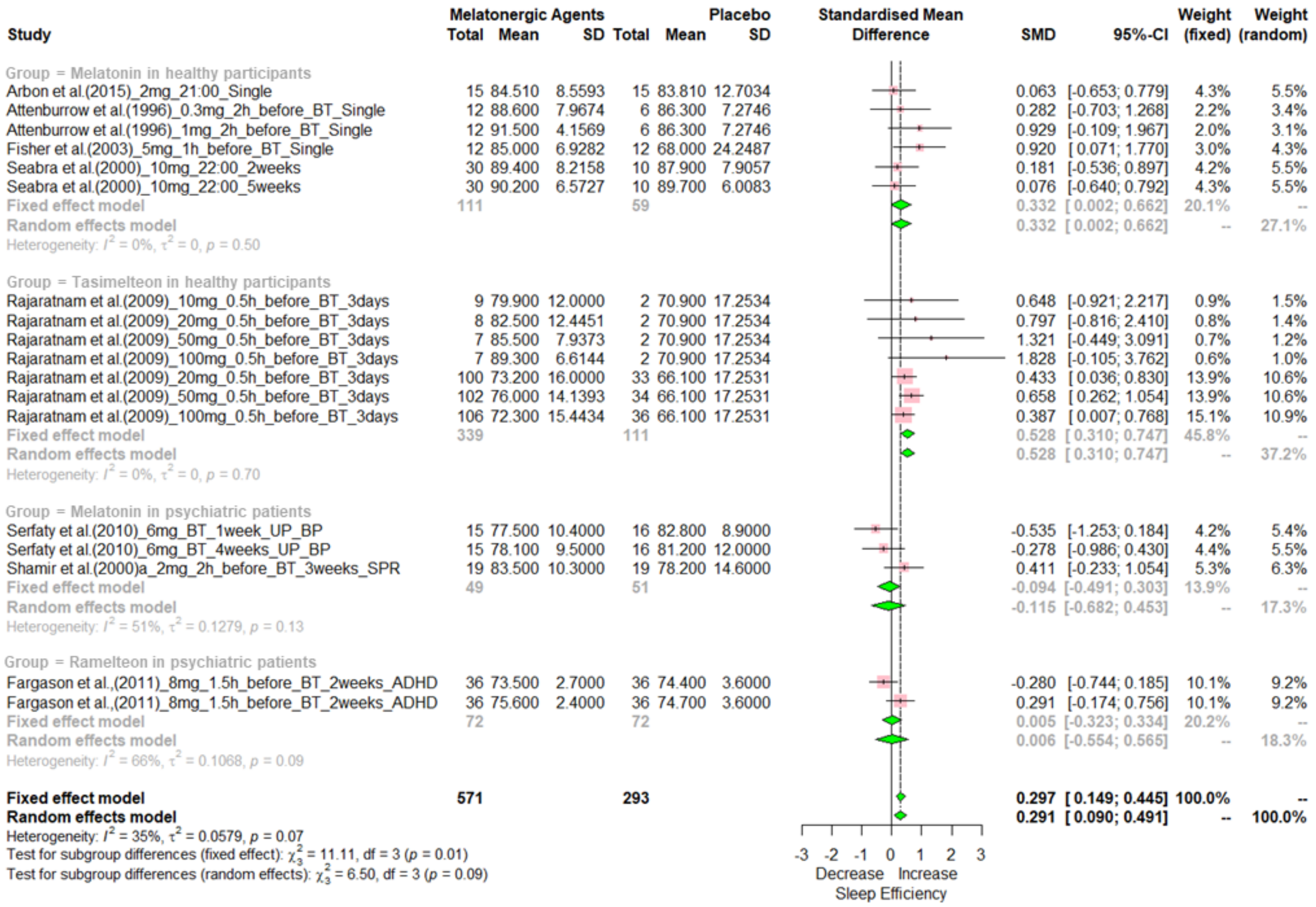

Supplementary Figure 4. Meta-ANOVA on efficacy of exogenous melatonin and melatonergic agents on sleep efficiency (SE) in healthy participants and psychiatric patients. The standardized mean difference (SMD) of 18 comparative datasets were synthesized. The pooled SMD in healthy participants showed that exogenous melatonin and tasimelton significantly increase SE compared to placebo (Exogenous melatonin: Heterogeneity I^2=0%, τ^2=0, p=0.50, fixed effect model *SMD [95% CI]* = 0.332[0.002 ~ 0.662]; Tasimelteon: Heterogeneity I^2=0%, τ^2=0, p=0.70, fixed effect model *SMD [95% CI]* = 0.528[0.310 ~ 0.747]). The pooled SMD in psychiatric patients showed that exogenous melatonin and ramelteon did not change SE compared to placebo (Exogenous melatonin: Heterogeneity I^2=51%, τ^2=0.1279, p=0.13, random effect model *SMD [95% CI]* = -0.115[-0.682 ~ 0.453]; Ramelteon: Heterogeneity I^2=66%, τ^2=0.1068, p=0.09, random effect model *SMD [95% CI]* = 0.006[-0.554 ~ 0.565]).

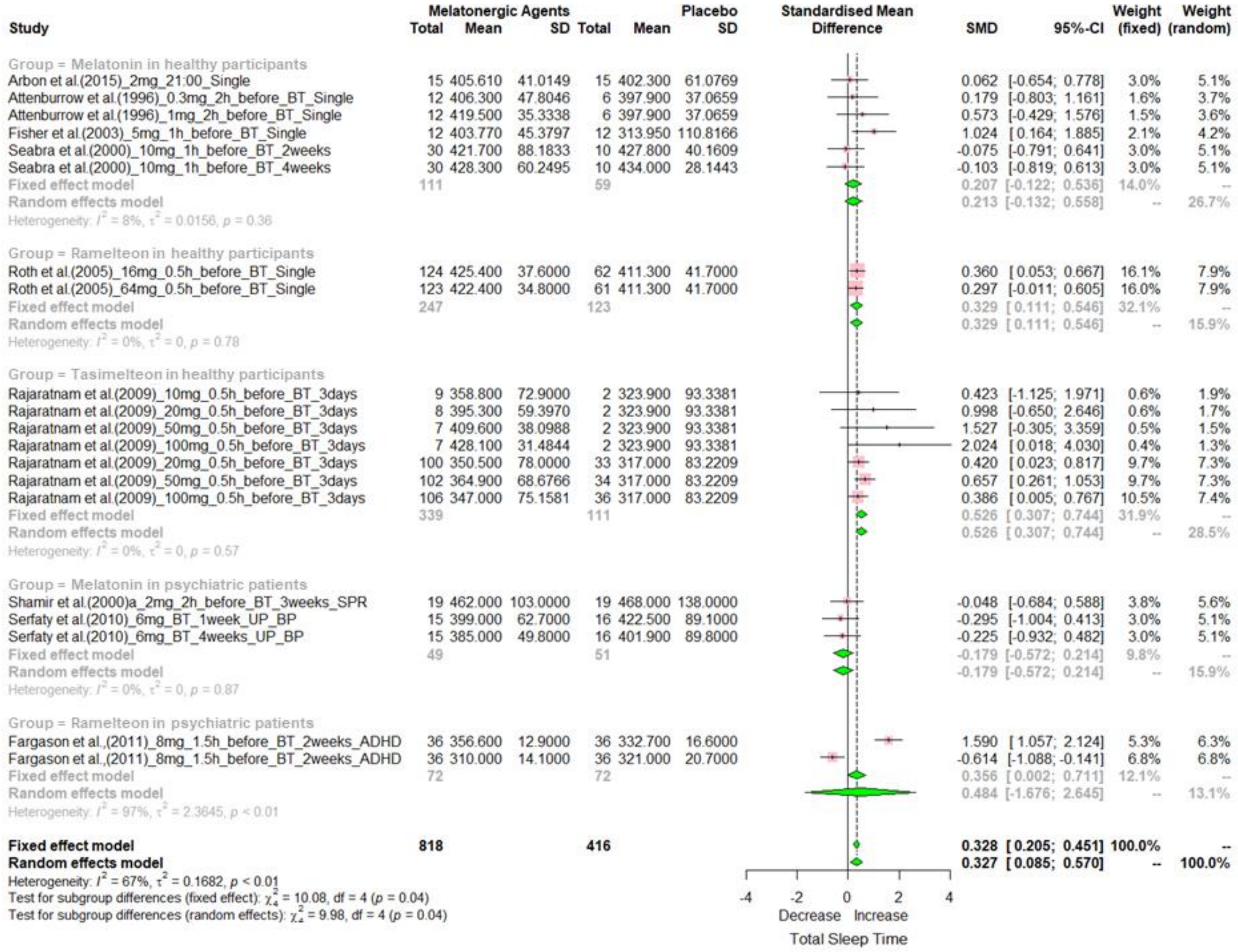

Supplementary Figure 5. Meta-ANOVA on efficacy of exogenous melatonin and melatonergic agents on total sleep time (TST) in healthy participants and psychiatric patients. The standardized mean difference (SMD) of 20 comparative datasets were synthesized. The pooled SMD in healthy participants showed that melatonergic agents significantly increase TST compared to placebo (Exogenous melatonin: Heterogeneity I^2=8%, τ^2=0.0165, p=0.36, fixed effect model *SMD [95% CI]* = 0.207[-0.122 ~ 0.536]; Ramelteon: Heterogeneity I^2=0%, τ^2=0, p=0.78, fixed effect model *SMD [95% CI]* = 0.329[0.111 ~ 0.546]; Tasimelteon: Heterogeneity I^2=0%, τ^2=0, p=0.57, fixed effect model *SMD [95% CI]* = 0.526[0.307 ~ 0.744]). The pooled SMD in psychiatric patients show that exogenous melatonin and ramelteon did not change TST compared to placebo (Exogenous melatonin: Heterogeneity I^2=0%, τ^2=0, p=0.87, fixed effect model *SMD [95% CI]* = -0.179[-0.572 ~ 0.214]; Ramelteon: Heterogeneity I^2=97%, τ^2=2.3645, p<0.01, random effect model *SMD [95% CI]* = 0.484[-1.676 ~ 2.645]).

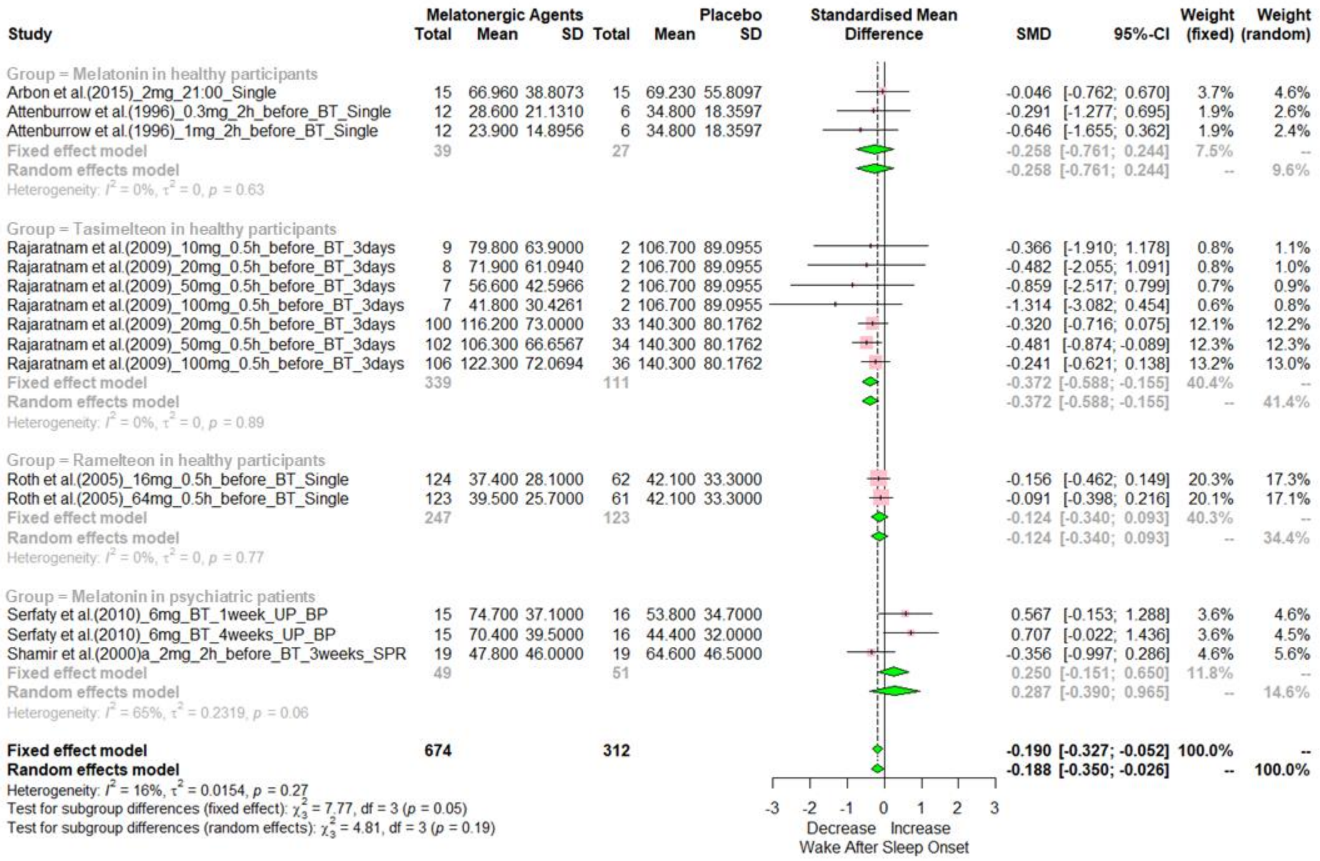

Supplementary Figure 6. Meta-ANOVA on efficacy of exogenous melatonins and melatonergic agents on wake after sleep onset (WASO) in healthy participants and psychiatric patients. The standardized mean difference (SMD) of 15 comparative datasets were synthesized. The pooled SMD in healthy participants showed that only tasimelteon significantly decrease WASO compared to placebo (Exogenous melatonin: Heterogeneity I^2=0%, τ^2=0, *p*=0.63, fixed effect model *SMD [95% CI]* = -0.258[-0.761 ~ 0.244]); Tasimelteon: Heterogeneity I^2=0%, τ^2=0, *p*=0.89, fixed effect model *SMD [95% CI]* = -0.372[-0.588 ~ -0.155]; Ramelteon: Heterogeneity I^2=0%, τ^2=0, *p*=0.77, fixed effect model *SMD [95% CI]* = -0.124[-0.340 ~ 0.093]). The pooled SMD in psychiatric patients showed that exogenous melatonin did not change WASO compared to placebo (Exogenous melatonin: Heterogeneity I^2=65%, τ^2=0.2319, *p*=0.06, random effect model *SMD [95% CI]* = 0.287[-0.390 ~ 0.965]).
